# Supplementary material for: Mesenchymal Stem Cells from COPD Patients Are Capable of Restoring Elastase-Induced Emphysema in a Murine Experimental Model
Source: Int J Mol Sci. 2023 Mar 18;24(6):5813. doi: 10.3390/ijms24065813 (PMC10054868; doi:10.3390/ijms24065813)
Supplement: Supplementary file 1 [file ijms-24-05813-s001.zip › ijms-2222607-supplementary.pdf]

Supplementary Materials: The following supporting information can be downloaded at: https://www.mdpi.com/article/10.3390/ijms24065813/s1, Figure S1: Administration of human MSC induced mild inflammation in immunocompetent but not in immunodeficient mice lungs; Figure S2: Intravenous (i.v.) and intratracheal (i.t.) are the most efficient routes to deliver human MSC in the mouse lung; Figure S3: Pre-stained AD-MSC signal is found distributed preferable in the lung when applied i.v. whereas it remains locally at the zone the i.p. application; Figure S4: Human mitochondria are found in the murine lungs after UC-MSC administrations.

Supplementary Figures

Supplementary Figure S1

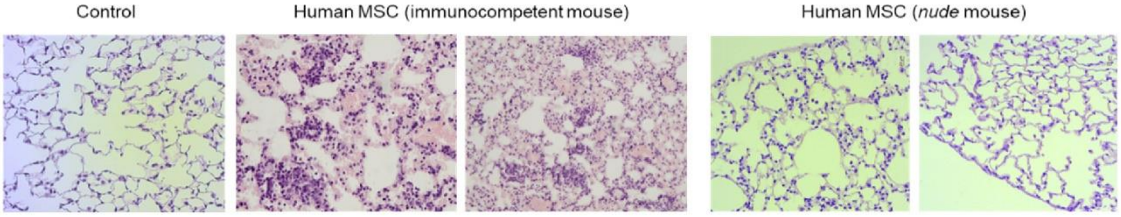

Supplementary Figure S1. Administration of human MSC induced mild inflammation in immunocompetent but not in immunodeficient mice lungs. Representative images of histological analysis of lung inflammation by H&E staining in wild type (immunocompetent) compared to immunodeficient (nude) mice. Mice were treated intratracheally with human MSC. Only PBS was applied in the control group.

Supplementary Figure S2

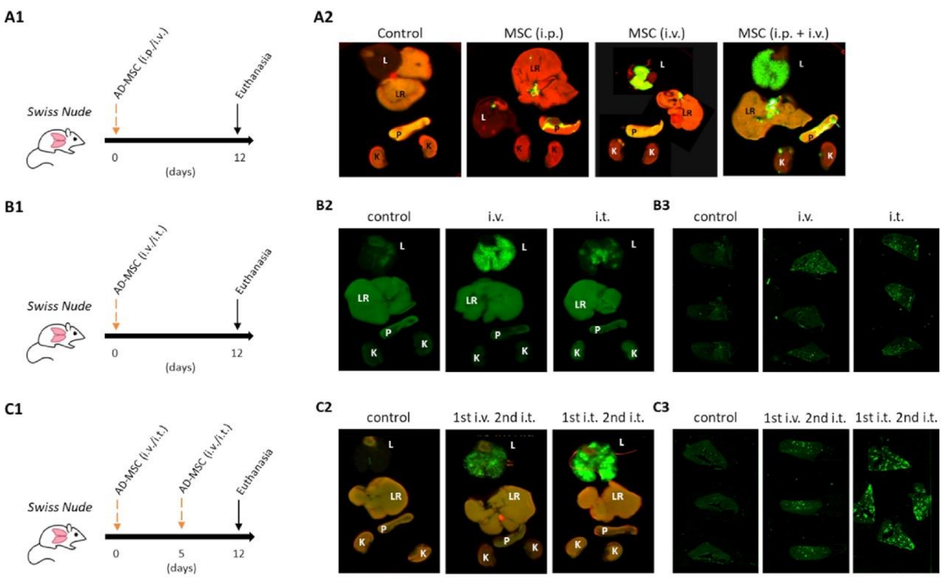

Supplementary Figure S2. Intravenous (i.v.) and intratracheal (i.t.) are the most efficient routes to deliver human MSC in the mouse lung. Assessment of the distribution of CellVue™ NIR815 pre-stained human AD-MSC delivered intraperitoneally (i.p.), intravenously (i.v.) or intratracheally (i.t.) at the timepoints indicated within whole organs; lung (L), liver (LV), pancreas (P) and kidneys (K). **(A)** CellVue™ NIR815 distribution within the mouse organs after i.p, i.v., and both (i.p + i.v.) administration. Chronogram **(A1)** and imaging of whole organs **(A2)**. **(B** and **C)** Comparison of the distribution of human CellVue™ NIR815 following i.v. and i.t administration routes as a single dose **(B),** or as two different doses **(C). B1** and **C1:** chornograms, **B2** and **C2**: imaging of whole organs, and **C3** and **C3:** histology of lung lobes.

Supplementary Figure S3

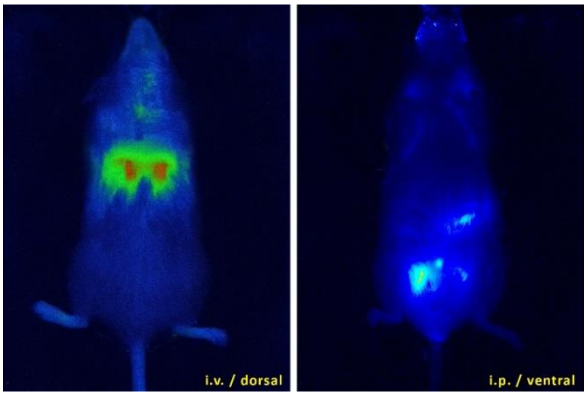

Supplementary Figure S3. Pre-stained AD-MSC signal is found distributed preferable in the lung when applied i.v. whereas it remains locally at the zone the i.p. application. Representative images of full animal bodies showing the fluorescent distribution of the signal from CellVue™ NIR815 pre-stained AD-MSC applied i.v. (left) or i.p. (right) at day 7 after administration. Abbreviations: intravenously (i.v.), intratracheally (i.t.).

Supplementary Figure S4

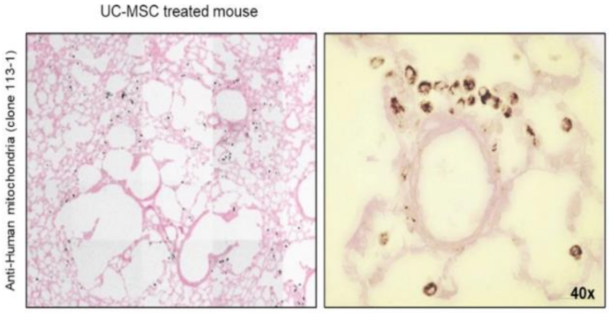

Supplemental Figure S4. Human mitochondria are found in the murine lungs after UC-MSC administrations. Staining of human mitochondria by immunohistochemistry in lung sections of UC-MSC treated mice after 4 days of i.t. of last cell administration.
